# Glutathione as a Biomarker in Parkinson's Disease: Associations with Aging and Disease Severity

**DOI:** 10.1155/2016/9409363

**Published:** 2016-06-30

**Authors:** Laurie K. Mischley, Leanna J. Standish, Noel S. Weiss, Jeannie M. Padowski, Terrance J. Kavanagh, Collin C. White, Michael E. Rosenfeld

**Affiliations:** ^1^Bastyr University Research Institute, 14500 Juanita Drive NE, Kenmore, WA 98133, USA; ^2^UW Graduate Program in Nutritional Sciences, 305 Raitt Hall, P.O. Box 353410, Seattle, WA 98195, USA; ^3^Department of Radiology, University of Washington (UW), P.O. Box 357115, 1959 NE, Pacific Seattle, WA 98195, USA; ^4^Department of Epidemiology, University of Washington (UW), 1959 NE Pacific Street, Health Sciences Building F-262, P.O. Box 357236, Seattle, WA 98195, USA; ^5^Elson S. Floyd College of Medicine and College of Pharmacy, Washington State University, P.O. Box 1495, Spokane, WA 99210-1495, USA; ^6^Department of Environmental & Occupational Health Sciences, University of Washington, P.O. Box 357234, Seattle, WA 98195, USA

## Abstract

*Objectives*. Oxidative stress contributes to Parkinson's disease (PD) pathophysiology and progression. The objective was to describe central and peripheral metabolites of redox metabolism and to describe correlations between glutathione (Glu) status, age, and disease severity.* Methods*. 58 otherwise healthy individuals with PD were examined during a single study visit. Descriptive statistics and scatterplots were used to evaluate normality and distribution of this cross-sectional sample. Blood tests and magnetic resonance spectroscopy (MRS) were used to collect biologic data. Spearman's rank-order correlation coefficients were used to evaluate the strength and direction of the association. The Unified PD Rating Scale (UPDRS) and the Patient-Reported Outcomes in PD (PRO-PD) were used to rate disease severity using regression analysis.* Results*. Blood measures of Glu decreased with age, although there was no age-related decline in MRS Glu. The lower the blood Glu concentration, the more severe the UPDRS (*P* = 0.02, 95% CI: −13.96, −1.14) and the PRO-PD (*P* = 0.01, 95% CI: −0.83, −0.11) scores.* Discussion*. These data suggest whole blood Glu may have utility as a biomarker in PD. Future studies should evaluate whether it is a modifiable risk factor for PD progression and whether Glu fortification improves PD outcomes.

## 1. Introduction

It is well established that redox stress contributes to PD pathophysiology and progression [1, 2]. Comprised of reduced (GSH) and oxidized (GSSG) forms, total glutathione (Glu) is essential for maintaining redox homeostasis, clearing metabolic waste, and serving as a reservoir for amino acids in the central nervous system (CNS). GSH deficiency was first hypothesized to play a causative role in PD in 1982 [3].* Post mortem* analysis of nigral tissue of individuals with PD exhibits deficiency of the antioxidant GSH compared to controls early in the course of disease [4, 5]. GSH deficiency is thought to incapacitate the cell's ability to metabolize cellular waste and impair defense against reactive oxygen species (ROS), reactive nitrogen species (RNS), and H_2_O_2_.

GSH deficiency has long been implicated in PD degeneration [6, 7], although little is known about human GSH or Glu concentrations in living humans with PD. It is not known whether peripheral measures of Glu status correlate with CNS Glu status. Recently, magnetic resonance spectroscopy (MRS) technology has evolved to permit the noninvasive estimation of regional CNS Glu concentrations [8], although ranges have not been described in a PD population. “Antioxidant Status” blood and urine panels are readily available. The goal of this study was to describe central and peripheral measures of Glu status in individuals with PD and evaluate whether Glu status was associated with PD severity.

PD research is in desperate need of a biomarker that predicts disease progression and can be targeted with the goal of modification and improved outcomes [9]. Any redox measure suggestive of age-related decline and/or associated with disease severity may have potential to serve an unmet need in PD research and practice.

## 2. Materials and Methods

**Figure 1:**
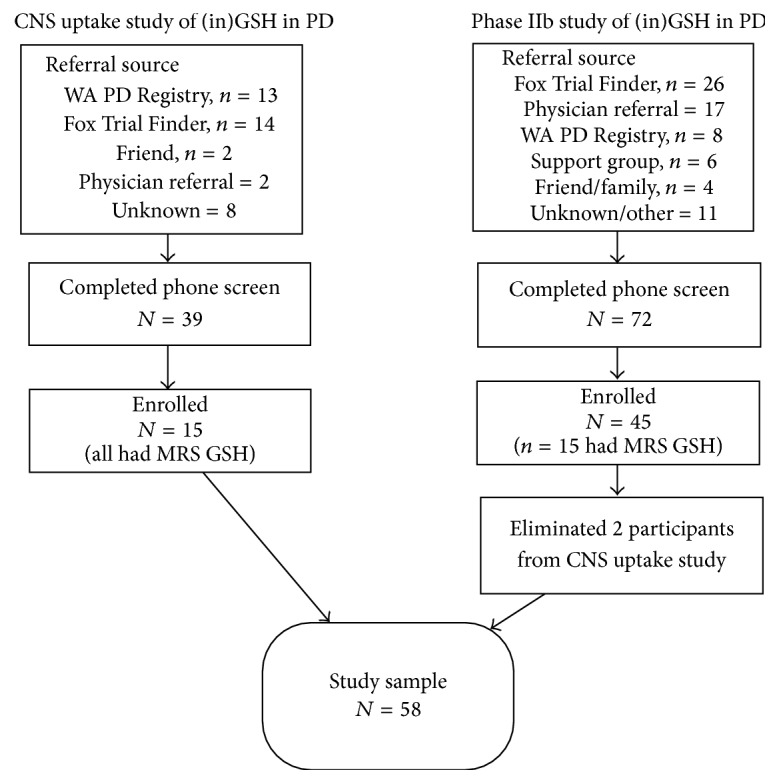
Enrollment Algorithm describing where participants from both studies were recruited and how the databases were merged for this study.

This is a combined dataset from two separate studies on topics related to PD and redox status. Blood samples for both studies were drawn within a year of one another and were all collected in Seattle, WA, by the same study staff. The demographics and referral sources were similar between the groups (Figure 1).

### 2.1. CNS Uptake Study of (in)GSH (*n* = 15)

This study was approved by the University of Washington (UW) IRB and listed on ClinicalTrials.gov prior to enrolling study participants. All participants were over 18 years of age, spoke English, had at least three of the required positive criteria for PD from Step 3 of the UK Brain Bank Diagnostic Criteria for PD, and had Hoehn and Yahr scores between 2 and 3. Due to the circadian rhythm of Glu, all urine samples collected were first morning samples, all MRI scans were scheduled at 8 am, and all blood collection occurred immediately following the scan, at approximately 9:45 am. All subjects were fasting at time of data collection.

### 2.2. Phase IIb Study of (in)GSH (*n* = 45)

This study was approved by the Bastyr University and UW IRBs and registered on Clinicaltrials.gov before the first participant was enrolled. As this was a longitudinal study, for this analysis only baseline measures were used. All participants had Hoehn and Yahr scores ranging from 1 to 3 and a diagnosis of PD made by a clinical neurologist in the previous 10 years. Due to the circadian rhythm of GSH, all urine samples were first morning samples. Blood draws were performed when the participant was in a nonfasting state. Participants were asked to maintain their regular diet but avoid large meals. There were two participants who enrolled in both studies; they were dropped from the second dataset.

For both studies, participants were invited to participate whether or not they were on dopaminergic medications. Both studies excluded individuals with a history of epilepsy, stroke, brain surgery, or structural brain disease, with the presence of other serious illnesses, pregnant or at risk of becoming pregnant, or with a history of sulfur sensitivity, for example, prior reaction to N-acetylcysteine (N-AC), methylsulfonylmethane (MSM), S-adenosyl methionine (SAMe), a recent history of asthma, supplementation with any form of Glu or Glu precursor (i.e., NAC) for six months prior to baseline study visit, current drug or alcohol use or dependence, or diagnosis of mental illness.

### 2.3. Laboratory Methodology

Total whole blood Glu, enzyme activity of superoxide dismutase, and Glu peroxidase were measured spectrophotometrically from RBC lysate using the Abbott Diagnostics Architect System (ADAS). Lipid peroxides were measured in serum and urine following hydrolysis and reaction with thiobarbituric acid. Malondialdehyde was used as the standard for the determination of concentrations of lipid peroxidation products.

Serum cysteine and cystine were measured using an adaption of the Gaitonde procedure [10] developed for the detection of amino acids which uses the colorimetric reaction of the amino acids with ninhydrin. Serum sulfate concentrations were determined using a turbidimetric assay which utilizes the chemical properties of sulfate ions to cause the formation of precipitate that can be measured by the absorbance of light using the ADAS. All tests above were performed by Genova Diagnostics (CLIA: #34D0655571, Asheville, NC, USA). GSH : GSSG ratios were measured at the University of Washington Department of Environmental and Occupational Health Sciences using previously described methods [11].

### 2.4. MRS Glu

A 3-Tesla Philips Achieva magnetic resonance imaging (MRI) scanner (Best, Netherlands) was used at the University of Washington Integrated Brain Imaging Center to obtain spectra with a 32-channel SENSE phased-array head coil. A cubic volume of interest (VOI), 4 × 4 × 5 cm, was centered on the left dorsal putamen at the level of the lentiform nucleus. This VOI was selected because of its relatively homogenous composition of neurons and astrocytes and suitable distance from bone and fluid, which could compromise signal quality. All scans were scheduled for 8 am.

### 2.5. Statistical Analyses

Scatterplots for* a priori* hypothesis were evaluated to look for trends in association and normality of distributions. Spearman correlation coefficients and regression analyses were determined using Stata (StataCorp, Stata/IC 13.1 for Mac). It is possible that the influence of increasing age on the risk of neurodegenerative disease is mediated in part by diminishing levels of GSH with increasing age. Therefore, analyses were performed with and without adjustment for age.

## 3. Results and Discussion

Two individuals enrolled in both studies, data obtained on that subject in the second study were discarded, reducing the total to *N* = 58. MRS data was discarded due to participant motion in scanner (*n* = 1) or Glu : creatine concentration in the brain was below the level of detection (*n* = 8).

Most participants resided in the Pacific Northwest, although a few traveled 5+ hours to participate. All but one participant was identified as Caucasian. Demographics and baseline disease severity are presented in Table 1.

**Figure 2:**
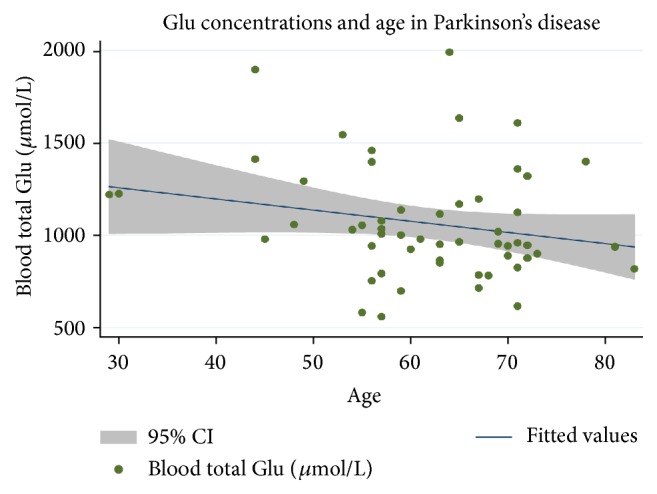
Cross-sectional analysis of whole blood total GSH concentrations in 58 individuals with PD (*r* = −0.2218).

To evaluate whether any of the GSH measures may reflect expected age-related physiological decline, scatterplots were drawn to evaluate GSH measures in a cross-sectional fashion. Whole blood Glu decreased with age (*r* = −0.2218), although MRS Glu : creatine and percent GSSG did not (*r* = −0.01 and *r* = 0.1263, resp.). Based on this sample, there is an estimated six-point decrease in micromol/L total GSH for each year of age (*P* = 0.104, 95% CI: −13.34, 1.27) (Figure 2).

Measurements of brain, blood, and urine redox measurements from individuals with PD are described. Data on % GSSG from Phase 2b were discarded due to high variability between results run in triplicate (Table 2).

**Figure 3:**
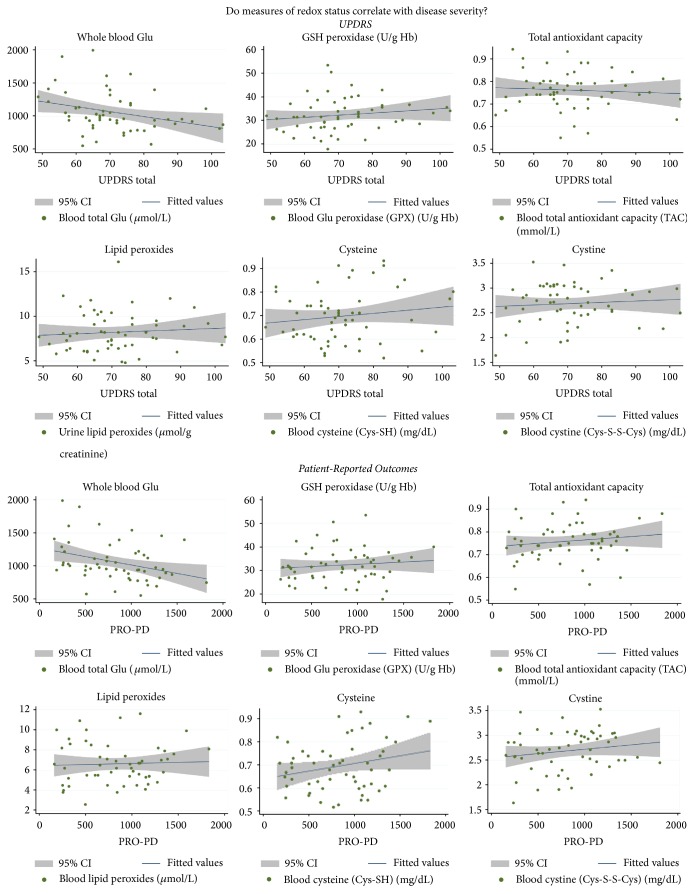
Scatterplot correlations between clinically available redox measurements and Parkinson's disease severity.

The UPDRS and PRO-PD were used to evaluate disease severity. In both scales higher scores represent more severe disease on both outcome measures used. There was a statistically significant correlation between whole blood total Glu levels and UPDRS score (*P* = 0.022, 95% CI: −13.96, −1.14). Linear regression analysis demonstrated a statistically significant correlation between PRO-PD scores and whole blood total Glu concentrations, with a 100-point increase in *μ*mol/L Glu being associated with a 47-point decrease in PRO-PD score (*P* = 0.01, 95% CI: −0.826, −0.119) (Figure 3).

The approximate midpoint for blood Glu was 1000 *μ*mol/L; an analysis was performed to evaluate whether individuals with Glu concentrations over 1000 *μ*mol/L had better PD outcome scores than those with Glu concentrations < 1000 (Table 3).

## 4. Conclusions

Of the panel of redox measurements evaluated here, whole blood Glu was the most sensitive to aging and the only measure that was associated with statistically significant severity of PD, as measured by UPDRS and the PRO-PD. The correlation between Glu concentrations and PRO-PD was stronger than that with the UPDRS, most likely because PRO-PD is more inclusive of nonmotor symptoms and does not fluctuate throughout the course of the day, and the slider bar design permits greater sensitivity to change.

The absence of well-established reference ranges, modest sample size (*n* = 58), and lack of control data are all limitations of this study. The strength of consistency between studies bodes well for the reliability of these clinically available biomarkers.

The lack of decline in MRS GSH with age or disease severity may have several explanations. First, eight of 30 participants had brain concentrations below the limit of detection of CNS GSH and were thus deleted from the dataset, which artificially drove up the mean. This could be statistically managed with appropriate* a priori* hypotheses in future studies (e.g., assign midpoint score between zero and lowest detectable).

Although MRS is capable of detecting increases in CNS GSH following (in)GSH administration, these data suggest MRS putamen concentrations cannot be extrapolated to reflect body status. In support, MRS GSH did not correlate with disease severity by either outcome measure; while MRS GSH may be useful for demonstrating target validation for augmentation efforts, MRS GSH does not appear to predict clinical status.

Of the assessments evaluated here, whole blood Glu was the most sensitive to aging and the only measure that was statistically associated with better PD status, as measured by UPDRS and the PRO-PD, suggesting it may have utility as biomarker for PD progression. Whole blood GSH is relatively stable when appropriately stored; banked samples from prior PD studies and ongoing study repositories should consider measuring Glu. The degree to which GSH is a modifiable risk factor in PD progression should be evaluated prospectively in an appropriately controlled study, giving consideration to dietary Glu intake.

## Figures and Tables

**Table 1:** Participant demographics.

	CNS uptake *n* = 15 Mean (SD)	Phase IIb *n* = 43 Mean (SD)	Combined *N* = 58 Mean (SD)
Gender			
Male	11 (73%)	22 (51%)	33 (57%)
Female	4 (27%)	21 (49%)	25 (43%)
Age, years	65.5 (11.2)	62.2 (10.8)	62.2 (10.8)
Disease severity			
Years since PD diagnosis	6.15 (6.24)	3.54 (2.2)	4.26 (3.9)
UPDRS	78.9 (15.4)	67.8 (9.5)	70.7 (12.2)
PRO-PD	869 (334)	804 (422)	821 (399)
Hoehn & Yahr			
1		9 (20.9%)	9 (15.5%)
1.5		6 (14.0%)	6 (10.3%)
2	6 (40%)	17 (39.5%)	23 (39.7%)
2.5	4 (27%)	7 (16.3%)	11 (19.0%)
3	5 (33%)	4 (9.3%)	9 (15.5%)

UPDRS: Unified Parkinson's Disease Rating Scale; PRO-PD: Patient-Reported Outcomes in Parkinson's Disease.

**Table 2:** Measures of brain, blood, and urine redox status in individuals with Parkinson's disease.

	CNS uptake	Phase 2b baseline	Combined
	*n* = 15	*n* = 43	*N* = 58
Brain Glu concentrations			
MRS Glu : creatine ratio (putamen) (*n* = 21)	0.026 (.018)	.030 (.017)	.028 (.017)
Whole blood			
Total GSH (micromol/L) (*n* = 55)	1027.64 (279.15)	1076.02 (311.85)	1063.71 (302.08)
RBC % GSSG	0.96%	—	—
Serum			
Sulfate (mg/dL) (*n* = 56)	3.83 (.62)	3.60 (.70)	3.66 (.69)
Cysteine (mg/dL) (*n* = 56)	.69 (.12)	.70 (.11)	.70 (.11)
Cystine (mg/dL) (*n* = 56)	2.78 (.34)	2.66 (.43)	2.69 (.41)
Lipid peroxides (micromol/L)	6.95 (2.49)	6.52 (1.87)	6.63 (2.03)
Total antioxidant capacity (mmol/L)	0.78 (0.07)	0.75 (0.084)	0.76 (0.08)
Urine			
Lipid peroxides (micromol/g cre)	8.43 (1.92)	8.14 (2.34)	8.22 (2.22)
8-OHdG (mcg/g creatinine) (*n* = 55)	9.86 (1.99)	9.92 (9.99)	9.91 (8.60)
Enzyme activity, whole blood			
Glu peroxidase (GPX) (U/g Hb)	34.64 (7.94)	31.55 (6.80)	32.34 (7.16)
Superoxide dismutase (U/g Hb)	11189 (4463)	14622 (4189)	13748 (4481)

**Table 3:** High versus low blood Glu concentrations in PD. In order to evaluate whether blood Glu may be associated with rate of progression, regression analysis is adjusted for age and years since PD diagnosis.

High versus low blood Glu in Parkinson's disease severity			
	GSH < 1000 *μ*mol/L	GSH ≥ 1000 *μ*mol/L	*P* (95% CI)
Unified PD Rating Scale (UPDRS)	75.82 (12.19)	65.9 (10.33)	0.087 (−11.90, 0.83)
Patient-Reported Outcomes in PD (PRO-PD)	1013.46 (365.37)	675.53 (381.57)	0.012 (−525.28, −67.49)
